# Processing spatial configurations in visuospatial working memory is influenced by shifts of overt visual attention

**DOI:** 10.1371/journal.pone.0281445

**Published:** 2023-02-09

**Authors:** J. David Timm, Frank Papenmeier

**Affiliations:** Department of Psychology, University of Tübingen, Tübingen, Germany; University of Zurich, SWITZERLAND

## Abstract

When memorizing multiple objects, humans process them in relation to each other, proposing a configuration benefit. Shifts in overt visual attention through eye movements might influence the processing of spatial configurations. Whereas some research suggests that overt visual attention aids the processing of spatial representations, other research suggests a snapshot-like processing of spatial configurations, thus likely not relying on eye movements. In the first experiment, we focused on the comparison between an enforced fixation and a free view condition regarding configurational effects. Participants encoded objects’ locations and were asked for changes at retrieval. One object was displaced in half of the trials and was either accompanied by a configuration or was displayed alone. In the second experiment, we expanded this idea by enforcing fixation during different task phases, namely encoding, maintenance and retrieval. We investigated if a fixed gaze during one specific phase drives the influence of eye movements when processing spatial configurations. We observed reliable configuration benefits for the free view conditions. Whereas a fixed gaze throughout the whole trial reduced the effect, enforced fixations during the task phases did not break the configuration benefit. Our findings suggest that whereas the processing of spatial configurations in memory is supported by the ability of performing shifts of overt visual attention, configurational processing does not rely on these shifts occurring throughout the task. Our results indicate a reciprocal relationship of visuospatial working memory and eye movements.

## Introduction

Single objects are remembered together with their surrounding objects in visuospatial working memory (VSWM), which is called a spatial configuration benefit [1]. Thus, a change of an object location is detected more easily, when the probed object is accompanied by the objects maintained in parallel than when the probed objects is shown alone at retrieval. Even when participants are instructed to memorize multiple single objects individually, the global spatial configuration of all encoded objects is processed automatically [1, 2]. Rendering a part of this global configuration relevant by a retro cue during maintenance results in similar configuration benefits for the relevant partial configurations [3–5]. Configuration benefits are not only limited to featureless objects [1, 2] but also arise with natural stimuli [6, 7]. While it is well established that spatial configurations are represented in VSWM, the conditions causing their representation are still not well understood. With the present research, we addressed this issue by studying the influence of eye-movements on the representation of spatial configurations in VSWM.

The performance and programming of saccadic eye-movements is strongly interconnected with visual attention and visuospatial working memory. For example, performing a saccadic eye-movement to one location inevitably causes the direction of covert visual attention to the very same location making it impossible to saccade to one target but to attend to another target [8, 9]. In a similar manner, limited working memory resources are directed towards saccade targets improving the precision of their representation in VSWM [10], with some research postulating that shifts in overt visual attention determine the composition of VSWM contents [11]. Vice versa, shifts of overt visual attention by means of saccadic eye-movements require an operating memory store for target selection, the maintenance of information across saccades, object correspondence, or gaze correction [12]. While the strong interconnection between saccadic eye-movements and VSWM has been shown for the representation of single objects, we extend this research by focusing on the relation between eye-movements and the representation of spatial configurations. We do so by first reviewing previous research on the relation between eye-movements and VSWM in general and thereafter focusing on the potential influence of eye-movements on the representation of spatial configuration in VSWM in particular.

Previous research established the relation between eye-movements and VSWM by either asking participants to explicitly perform task-unrelated eye-movements [13] or by preventing participants from performing potentially task-related eye-movements due to fixation instructions [14–16]. For example, when participants concurrently memorize spatial information and try to detect a shape change of a visual object on the screen, memory for the spatial information is reduced when the shape is moving, thus causing participants to perform task-unrelated voluntary eye-movements, rather than stationary [13]. It is assumed that task-unrelated voluntary eye-movements interfere with the maintenance of spatial information [13]. The role of eye-movements for maintenance in VSWM was further investigated by instructing participants to either free view during maintenance or to fixate a specific location such as the fixation cross during maintenance [14–16]. The free-view eye-movement patterns during maintenance suggest that participants utilize eye-movements for rehearsal during maintenance by fixating previously occupied target locations [14–16]. Whereas asking participants to fixate the task-unrelated fixation cross instead of free-viewing reduces memory performance [14, 15], giving participants an instruction to fixate a single self-chosen location instead of free viewing during maintenance causes no reduction of memory performance [16]. The latter finding was attributed to participants still being able to rehearse the object locations with covert visual attention. This idea is supported by research showing that forcing covert visual attention to the fixation cross by the presentation of a dual-task during maintenance causes similar reductions in memory performance as instructing participants to actually fixate the fixation cross [14, 15]. It is important to note, however, that while instructions to fixate the fixation cross cause a general drop in memory performance, the specific means of the functional role of free view eye-movements during maintenance are still not well understood. For example, while fixation during maintenance causes a general drop in memory performance, it does not seem to increase the rate of time-based forgetting due to decay [14], and the fixation of objects during maintenance that undergo a change during test also does not necessarily cause better change detection for those objects [15].

Regarding the potential influence of eye-movements on the representation of spatial configuration in VSWM, it seems useful to first consider the process underlying the representation of inter-object relations in VSWM. Two competing accounts explaining the storage and processing of spatial configurations in VSWM can be identified in the literature. One account assumes a rather rigid, snapshot-like, representation of spatial configurations in VSWM whereas the other account assumes a more flexible representation.

The snapshot account is based on the observation that only the global spatial configuration of all encoded objects but not a partial spatial configuration of a subset of encoded objects results in a memory benefit [1, 2] and this snapshot might be represented in a separate view-dependent snapshot store in VSWM that is also used for spatial navigation [2, 17]. This idea fits with the finding that spatial configurations are represented in a view-dependent manner with configuration benefits disappearing when viewpoint changes of 30 or 60 degrees between encoding and retrieval are introduced [7]. Further, location change detection is impaired by changes to task-irrelevant features that destroy the perceptual grouping of the memorized objects [18].

The flexible account suggests that spatial configurations are stored interdependently in VSWM. It is based on the idea that the representation of inter-object relations such as summary statistics (mean color or location across individual object representations) might result from a hierarchical representation of features in VSWM with individual objects being organized in higher order hierarchical clusters [19–22]. This more flexible account is in accordance with recent findings showing that the influence of inter-objects relations on memory representations can be manipulated by shifting visual attention with retro cues during maintenance and thus after encoding has already been completed. This accounts for both the representation of object features such as orientation [23] and object locations as represented in spatial configurations [3–5]. Regarding the latter, it was shown that the presentation of valid retro cues during encoding caused a reorganization of spatial configurations to the relevant probed one [3–5].

Whereas the snapshot account suggests a rather holistic representation of spatial configurations, the flexible account emphasizes the role of individual objects as nodes for the higher-order representations. Applying both accounts to the potential role of eye-movements on the representation of spatial configurations in VSWM, on might predict that eye-movements are rather unimportant or even harmful for the representation of spatial configurations based on the snapshot account. That is, the spatial configuration benefit should be stronger for a fixation condition than for a condition with free eye-movements. Based on the flexible account, in contrast, one might predict that eye-movements and thus the shift of overt (and covert) attention might have a strong influence on the representation of individual objects and thus potentially also their inter-object relations. Thus, the spatial configuration benefit should be stronger with free eye-movements than in a fixation condition. There is some initial evidence supporting both views. In accordance with the former view, the implicit learning of spatial configurations within the contextual cueing paradigm is not only possible but even superior without eye-movements than with eye-movements [24]. In support of the latter view, an experiment that was designed to test the influence of retro cues and set-size on the reorganization of spatial configurations but that also manipulated eye-movements observed a configuration benefit for the group of participants allowed to perform eye-movements freely but not for the other group of participants being enforced to maintain fixation [3].

In our research, we focused on the role of eye-movements for the representation of spatial configurations on VSWM. That is, we were interested under which conditions the spatial inter-object relations between objects are utilized for VSWM, such that a location change is easier detected during test when the probed object is not presented alone but together with the global spatial configuration of all objects. There is prior related work that studied the memory for object locations within a spatial grid [14, 25, 26]. They showed that the presence of the spatial grid during maintenance supports the rehearsal of object locations whereas the absence of the spatial grid results in decay. Interestingly, a fixation instruction as compared with a free view instruction resulted in a general drop of memory performance that was stronger for the conditions with the spatial grid than without the spatial grid [14]. Nonetheless, the benefit of the presence of the spatial grid remained even with the fixation instruction. It is important to note, however, that there are some important differences between this prior work on environmental support during maintenance [14] and our present experiments. Our research focused on the representation of inter-object relations between memorized objects whereas this prior work focused on the influence of environmental support (spatial grid of target and distractor locations) on rehearsal during maintenance. Therefore, our manipulation of the spatial configuration addressed the presence of inter-object relations during test whereas the research on environmental support addressed the presence of a spatial grid during maintenance. This makes it difficult to draw clear predictions for our present experiments based on this prior work.

With the present set of two experiments, we aimed at investigating the role of eye-movements for the processing of spatial configurations in VSWM. We did so by manipulating eye-movements within-participants rather than between-participants [3] providing a stronger test of whether eye-movements rather than other random cognitive differences across groups of participants drive the encoding of spatial configurations. Further, we manipulated eye-movements in a fine-grained manner such as the encoding, maintenance and retrieval phase in Experiment 2 in order to shed light on the interrelation of eye-movement performance and the representation of spatial configurations in VSWM.

## Experiment 1

With this experiment, we investigated the influence of shifts of overt visual attention on the processing of spatial configurations in VSWM. Therefore, we manipulated whether participants had to maintain fixation or whether there were allowed to perform eye-movements freely and measured the benefit of the presence of configurational information during retrieval on memory performance in a location change detection task.

### Method

We performed the method including sample size and analyses as we had preregistered on OSF: https://osf.io/gw7v5.

### Participants

We used the R-Package powerbydesign [27] to conduct a power analysis based on our previous experiments [3, 4]. Our goal was to obtain at least a power of .90 at the standard of .05 alpha error probability. This led to a sample size of 56. Participants were invited for this experiment receiving course credit or monetary compensation. We paid 2€/15 min. We preregistered the following exclusion criteria: Participants identified as not doing the task (pressing always the same button or a performance level that does not deviate from chance) were supposed to be removed from the data set and replaced by new participants as well as any participants not completing the whole experiment. Eight participants had to be replaced due to a performance level that did not deviate from chance. Participants had normal or corrected-to-normal vision and their age ranged from 18 to 31 years (*M* = 23.9 years, *SD* = 3.3 years). Up to two participants were tested at the same time on different laptop computers. Simultaneous testing and monetary compensation were consistent throughout all experiments. The research was conducted in accordance with APA standards for ethical treatment of participants and with approval of the institutional review board of the University of Tübingen. All participants provided written informed consent.

### Materials

We presented eight grey squares (RGB color hex code: #777777) on a white background (#FFFFFF) on a 15.6” computer screen (Dell Precision M4800) using an SMI iView RED250 mobile eye tracker to record eye movements with a sampling rate of 250 Hz. The experiment was programmed with PsychoPy 1.85.6 [28]. Each square measured 0.8° x 0.8° (degrees of visual angle). The objects could appear in a 18° x 18° centered array (black outline: #000000). A black fixation cross (0.5° x 0.5°, color: #000000) was presented in the center of this array. We generated random object locations for each trial with a minimum center-to-center distance of twice the diameter of a square and with a minimum distance to the center of the array of once the diameter of a square. Participants’ heads were positioned in a chin rest.

### Procedure

**Fig 1:**
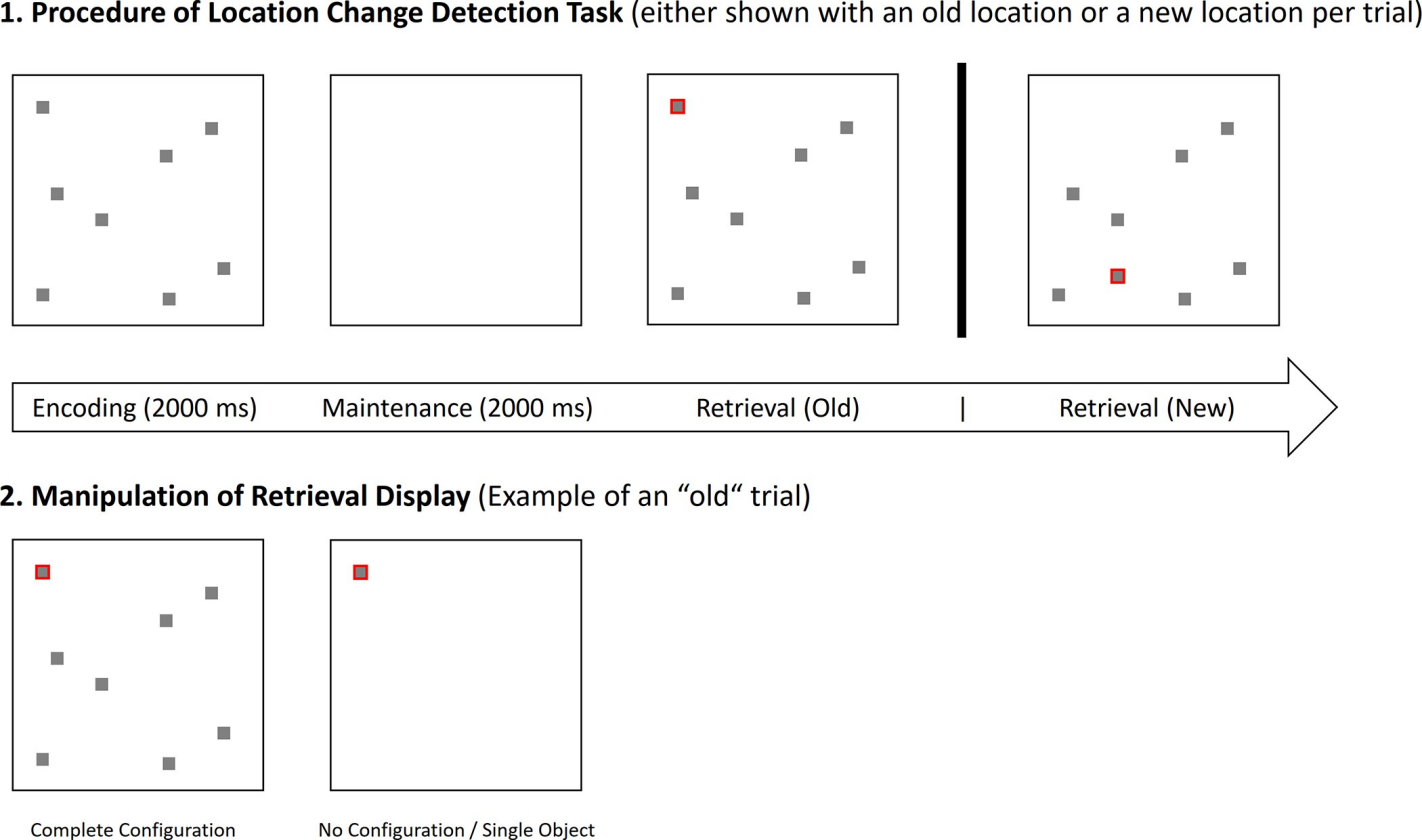
Location change detection task. Note that the fixation cross is not displayed in this figure for illustrative purposes.

Participants performed a location change detection task (see Fig 1). During encoding all objects were shown for 2000 ms. Then a maintenance phase of 2000 ms followed with no objects visible. At retrieval objects reappeared either with a configuration (global) or without configuration (single) and one object was marked by a red outline (#FF0000). We manipulated the position of the object probed at retrieval (new/old). Participants had to press the respective keyboard button (1/9) to indicate whether the object probed changed its location or not.

Trials were presented in randomized order with the restriction that the experiment consisted of two blocks containing 80 trials each leading to 160 trials in total. One block was performed without any restrictions regarding eye movements while the other block was performed with a fixed gaze. Participants started each trial with fixating the centric cross for 250 ms, which was visible across the whole trial. If participants did not hold the fixation throughout the trial in the enforced fixation condition, the trial was aborted and repeated at the end of the block. In detail, if gaze samples were recorded outside of an invisible surrounding circle with a radius of 1.5° around the centric cross for more than 250 ms, the respective enforced fixation trial was aborted and repeated at the end of the block. Within the enforced fixation block, a mean of 22.6% (*SD* = 14.6%) of the presented trials were aborted and repeated across participants. We counterbalanced the block order leading to one group of participants with a first block of free view and a subsequent block with a fixed gaze and vice versa for the other half of the participants. Each other condition occurred equally often within each block and, thus, also within the whole experiment. Participants performed an eye movement specific practice block, containing one trial per possible condition (4 trials), in the beginning of the experiment depending on the eye movement condition (with/without). In the beginning of the second block, another practice block was done, containing the other eye movement condition. Participants were not aware of a change in the possibility of shifting overt visual attention until that second practice block occurred. The whole experiment duration was approximately 45 minutes.

## Results and discussion

The data and the analysis can be obtained from https://osf.io/rqejx/. We performed the analyses in accordance with our preregistration. We calculated sensitivity (according to signal detection theory) as dependent measure across the responses to the old and new probe location trials [29]. Sensitivity d’ is defined as $d^{\prime }{=\Phi }^{-1}(phits)-\Phi^{-1}(pfa)$ with phits being the proportion of hits and pfa being the proportion of false alarms [29]. Hits refer to the accurate detection of old locations, and false alarms refer to responding “old” to a new location. Note that sensitivity cannot be calculated for either phits or pfa having values of 0.0 and 1.0. Thus, we corrected such values to the proportions equaling half a trial correct or half a trial incorrect respectively. Trials with response times exceeding 10 seconds were removed before the analysis (0.08%).

**Fig 2:**
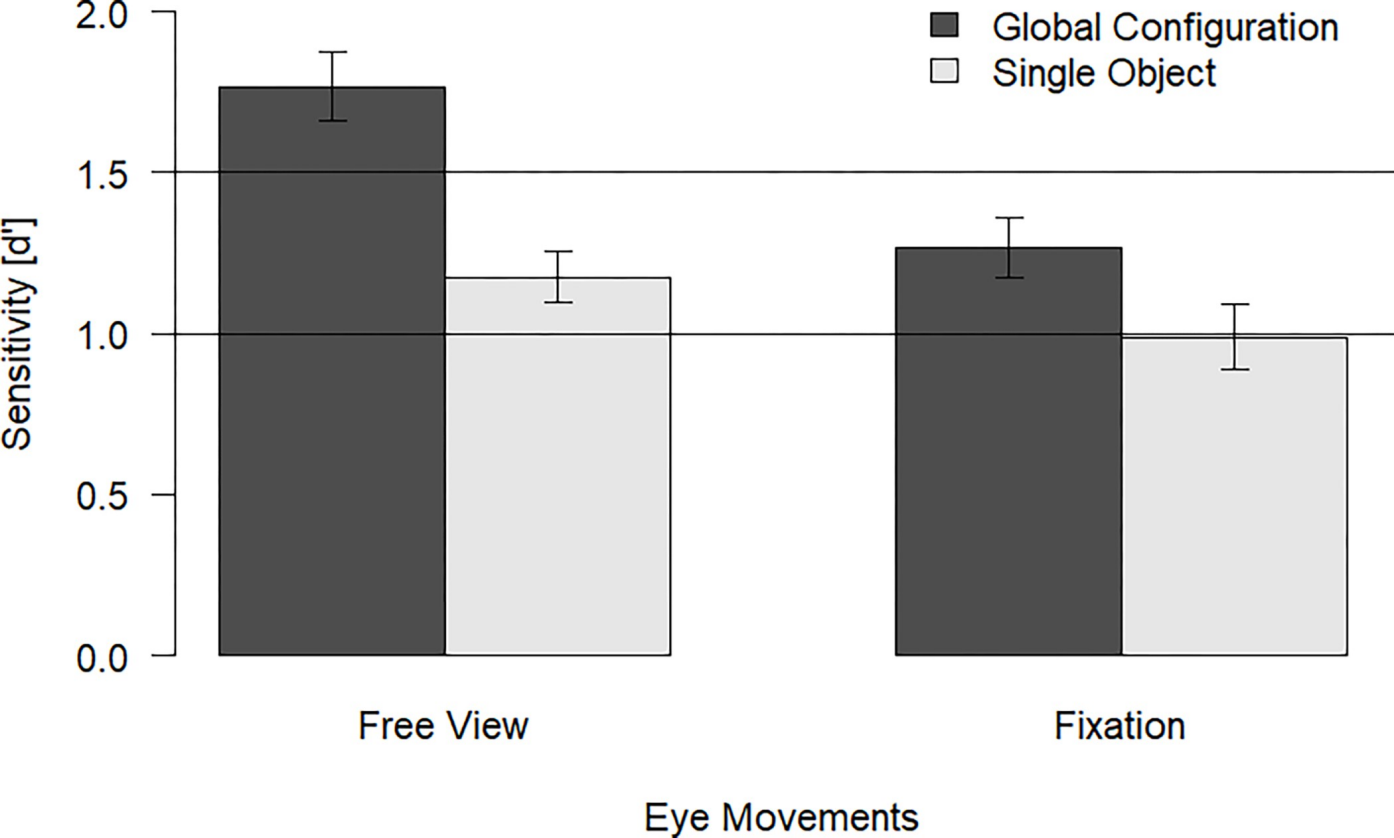
Sensitivity (d’) across conditions for Experiment 1. Error bars indicate the standard error of the mean (SEM).

We compared location change detection performance as indicated by the sensitivity measure across conditions using a 2 (eye movements: free view, fixation; within) x 2 (configuration: global, single; within) repeated-measures ANOVA (see Fig 2). Importantly, there was a significant interaction effect of eye movements and spatial configuration, *F*(1, 55) = 7.30, *p* = .009, η_p_^2^ = .12. That is, the configuration benefit was stronger for the free view trials than for the trials with enforced fixation. This suggests that the ability of planning and performing shifts of overt visual attention by eye movements supports the processing of spatial configurations. Furthermore, there was a significant main effect for eye movements *F*(1, 55) = 15.74, *p* < .001, η_p_^2^ = .22 and a significant main effect for configuration, *F*(1, 55) = 44.24, *p* < .001, η_p_^2^ = .45. Due to the interaction effect, we further investigated the conditions with t-tests (see Table 1).

**Table 1:** p-values–t-tests for configurations in Experiment 1 across conditions.

Eye Movements		Free View Global	Single	Fixed Gaze Global
Free View	Single	< .001*	---	---
Fixed Gaze	Global	< .001*	. 364	---
	Single	< .001*	.088	.003*

* statistically significant (p < .008, that is significance level of .05 Bonferroni-corrected for six post-hoc comparisons)

To investigate the influence of block order, we performed an additional exploratory mixed ANOVA with the factors eye movements (free view, fixation; within), configuration (global, single; within) as well as the group factor block order (fixation/free view, free view/fixation; between). The main effects of spatial configuration, *F*(1, 54) = 43.92, *p* < .001, η_p_^2^ = .45, and eye movements, *F*(1, 54) = 15.65, *p* < .001, η_p_^2^ = .22, as well as their interaction, *F*(1, 54) = 7.39, *p* = .009, η_p_^2^ = .12, remained significant. Importantly, neither the main effect of block order, *F*(1, 54) = 0.51, *p* = .480, η_p_^2^ = .01, nor any interaction effect including block order, all *F*(1, 54)s ≤ 1.67, *p*s ≥ .202, were significant. Therefore, block order did not influence our results.

Within the preregistration of both of our experiments we wrote that we would compare fixation and saccade parameters between hit and false alarm trials in the free view condition. This analysis is not informative for the present research question, but for the sake of completeness we present it within S1 Appendix.

To summarize, the overall ability in performing eye-movements (as compared with enforced fixations) had a significant effect on the processing of spatial configuration. The configuration benefit was larger under the free view condition than under enforced fixation. However, we still observed a configuration benefit also with enforced fixation. This speaks against the strong assumption of the configuration benefit depending on eye-movements. Rather, participants utilized the global spatial configuration of the objects for improving their memory performance and being able of perform free eye movements enhanced the processing of spatial configurations in VSWM.

## Experiment 2

With our first experiment, we investigated the influence of overt visual attention on the processing of spatial configurations in VSWM. As predicted, the configuration benefit was stronger in the free view trials than with enforced fixation. What remained unresolved, however, was whether it was the general ability in performing free shifts of overt visual attention or rather the free distribution of overt visual attention during specific task phase, such as during encoding, maintenance, or retrieval, that supported the processing of spatial configurations. Previous research suggests that eye-movements during specific phases, such as during maintenance [13–15], might aid memory performance. That is, participants tend to move their eye to previously encoded locations during maintenance, and fixating the fixation cross during maintenance reduces memory performance [14, 15]. Therefore, in contrast to Experiment 1, we did not enforce fixation throughout the whole trial in our Experiment 2, but we rather enforced fixation only during specific phase (i.e. encoding, maintenance, retrieval). By doing so, we investigated whether enforcing fixation in just one of the three phases might be enough to reduce the configuration benefit as compared with a free view condition.

### Method

We performed the method including sample size and analyses as we had preregistered on OSF: https://osf.io/vsfzk.

### Participants

We increased the desired sample size to 60 participants, because this allowed us to counterbalance block order across participants. We preregistered the following exclusion criteria: Participants identified as not doing the task (pressing always the same button or a performance level that does not deviate from chance) were supposed to be removed from the data set and replaced by new participants as well as any participants not completing the whole experiment. Nine participants had to be replaced due to a performance level that did not deviate from chance in the baseline condition. Participants had normal or corrected-to-normal vision and their age ranged from 19 to 36 years (*M* = 24.2 years, *SD* = 4.0 years). All participants provided written informed consent.

### Materials

We used the same materials with a 15.6” computer screen (Dell Precision M4800) and an SMI iView RED250 mobile eye tracker to record eye movements with a sampling rate of 250 Hz.

### Procedure

Participants performed the same location change detection task as in Experiment 1. Thus, participants again started each trial with fixating the centric cross for 250 ms, which was visible across the whole trial. We manipulated the possibility of performing eye movements. Participants performed a free view condition as in Experiment 1 (baseline) as well as three conditions in which they had to maintain fixation in one of the three task phases (encoding, maintenance, retrieval). For example, participants had to fixate the center of the screen during encoding and then had to free view during maintenance and retrieval. All participants performed the baseline condition as the first experimental block. The three fixation conditions were presented counterbalanced across participants. We controlled that participants fixated the centric cross in the respective phase and that they looked away from the fixation cross at least once for each free view phase forcing a saccade. If participants did not hold the fixation throughout the specific fixation phase, the trial was paused, and the screen turned black with a red cross (#FF0000). Participants were instructed that a red color of the cross indicates they had to regain the fixation and if executed, the trial was continued. The trial was paused when the fixation loss exceeded 100 ms. To avoid that participants fixated the center throughout the whole trial, we applied the same logic the other way around to the respective free view phases. If participants fixated the centric cross at the beginning of each free view phase for longer than 300 ms, the trial was paused, and the screen turned black with a blue cross (#0000FF). Participants were instructed that a blue color of the cross indicates they had to move their eyes away from the cross and if executed, the trial was continued. The proportion of paused trials and pause durations are presented within the table in S2 Appendix. Please note that there was a programming error in the experiment for the experimental blocks with enforced fixation during maintenance and enforced fixation during retrieval. For those blocks, the free view encoding phase was not paused as intended. That is, the blue cross correctly appeared as described above if participants fixated the centric cross at the beginning of the encoding phase for longer than 300 ms. However, the encoding phase was not prolonged by the presentation time of the blue cross as intended. Thus, participants saw the objects during encoding for at least 300 ms and up to 2000 ms depending on how long they held their fixation at the beginning of the encoding phase. The whole experiment duration was approximately 60 minutes.

## Results and discussion

The data and the analysis can be obtained from https://osf.io/h5yng/. We performed the analyses in accordance with our preregistration. We calculated sensitivity (according to signal detection theory) as in Experiment 1. Trials with response times exceeding 10 seconds were removed before the analysis (1.77%).

**Fig 3:**
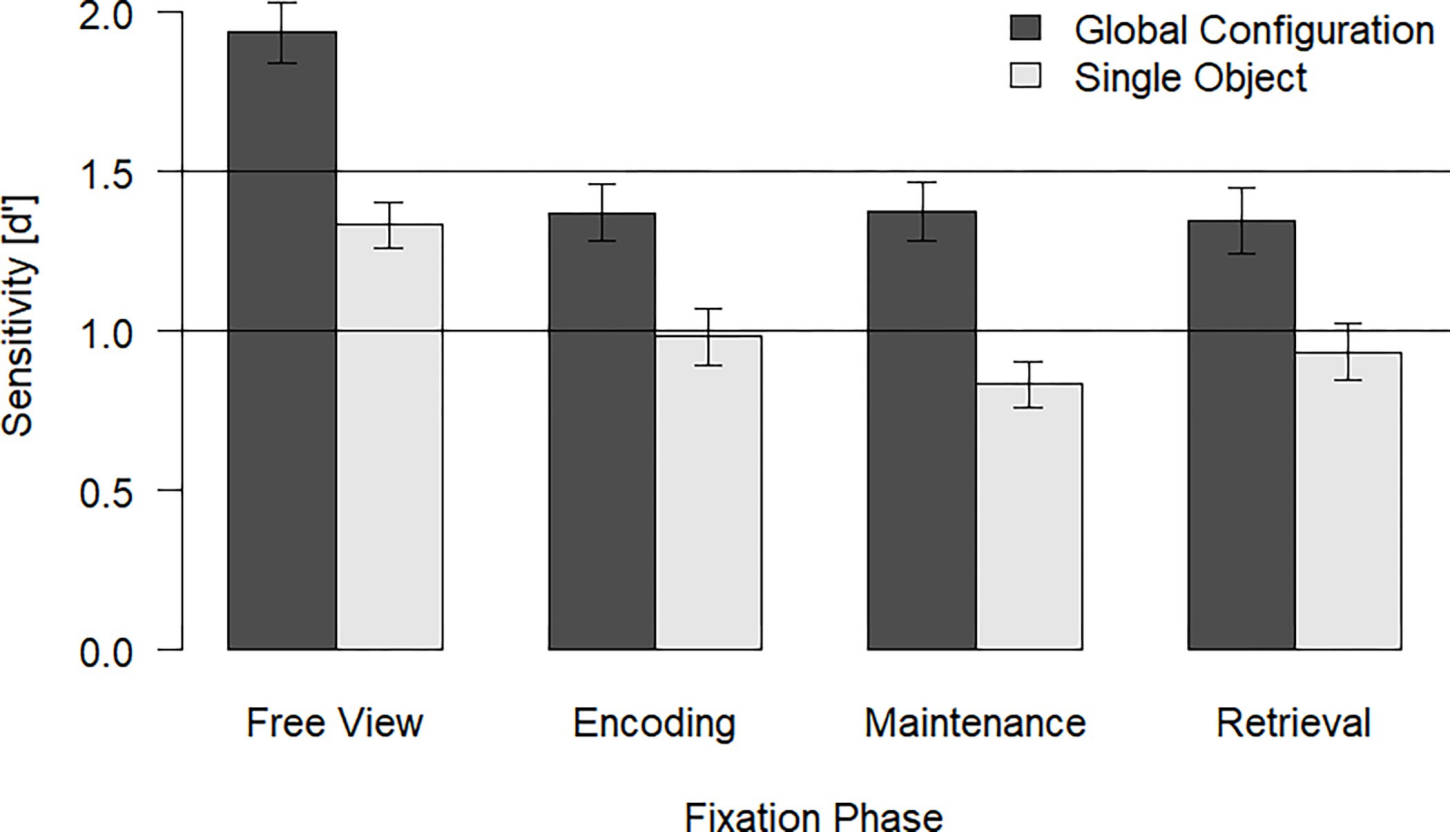
Sensitivity (d’) across conditions for Experiment 2. Error bars indicate the standard error of the mean (SEM).

We compared location change detection performance as indicated by the sensitivity measure across conditions using a 2 (configuration: global, single; within) x 4 (fixation phase: baseline, encoding, maintenance, retrieval; within) repeated-measures ANOVA. Strikingly, we did not observe a significant interaction of eye movement condition and configuration, *F*(3, 177) = 1.26, *p* = .288, η_p_^2^ = .02 (see Fig 3). Thus, enforcing fixation during specific task phases was not enough to reduce the configuration benefit as compared with the free view condition. This suggests that it is rather the general ability of planning and performing shifts of overt visual attention by eye movements that support the processing of spatial configurations in VSWM (see Experiment 1) than the performance of eye movements during specific task phases (present experiment). Furthermore, there were significant main effects for fixation phase, *F*(3, 177) = 25.94, *p* < .001, η_p_^2^ = .31, and configuration, *F*(1, 59) = 94.40, *p* < .001, η_p_^2^ = .62.

In an exploratory analysis, we collapsed across the data of all three conditions with enforced fixation and analyzed sensitivity with a 2 (configuration: global, single; within) x 2 (eye movements: free view, fixation in one phase; within) repeated-measures ANOVA. The interaction between eye movements and configuration remained non-significant, *F*(1, 59) = 2.34, *p* = .132, η_p_^2^ = .04. Furthermore, there were significant main effects for the factors eye movements, *F*(1, 59) = 87.82, *p* < .001, η_p_^2^ = .60, and configuration, *F*(1, 59) = 78.30, *p* < .001, η_p_^2^ = .57. Thus, the pattern of results remained the same no matter whether the three conditions with enforced fixation were considered separately or as one common condition.

### Exploratory analysis: Distance of probed object to center of the screen

Our experiments were designed to study the influence of overt visual attention on the processing of spatial configurations. Thus, we manipulated participants’ eye movements using the enforced fixation and free view instructions. Our experiments were, however, not designed to disentangle the influence of overt and covert visual attention on the processing of spatial configurations. One could argue that our free-view conditions also elicited shifts of covert visual attention, because the performance of eye-movement is strongly related to shifts of covert visual attention to the same location [8, 9]. Importantly, however, the reverse is not true. That is, enforcing overt visual attention to the center of the screen does not prevent participants from performing covert shifts of visual attention. Instead, it is well known that observers can perform covert shifts of visual attention during enforced fixation [30]. Research designed to control not only for the location of overt attention but also for covert attention often uses dual tasks to force covert visual attention to specific spatial locations, for example by requiring participants to constantly monitor those locations for specific stimuli [14, 15, 31]. Because we did not employ such a dual task, participants were free to move their covert visual attention within their visual field in our experiments. Thus, it remains possible that participants used shifts of covert visual attention to encode the spatial locations of the objects in our experiments, even when we enforced participants overt visual attention to the center of the screen. Because the spatial resolution of visual attention decreases with eccentricity [32], shifts of covert visual attention should be the less efficient in supporting the memory for object locations the further away the objects are from the center of the screen. In order to further investigate this potential influence of covert visual attention on our results, we performed an exploratory analysis investigating the influence of the distance of the probed object to the center of the screen on memory performance across the experimental conditions of Experiment 1 and 2. Whereas the conditions with enforced fixations, in particular across the whole trial (Experiment 1), were most informative for this exploratory analysis, we also included the free-view conditions into this analysis to provide a baseline on what influence distance might have for our stimuli regardless of fixation instructions.

**Fig 4:**
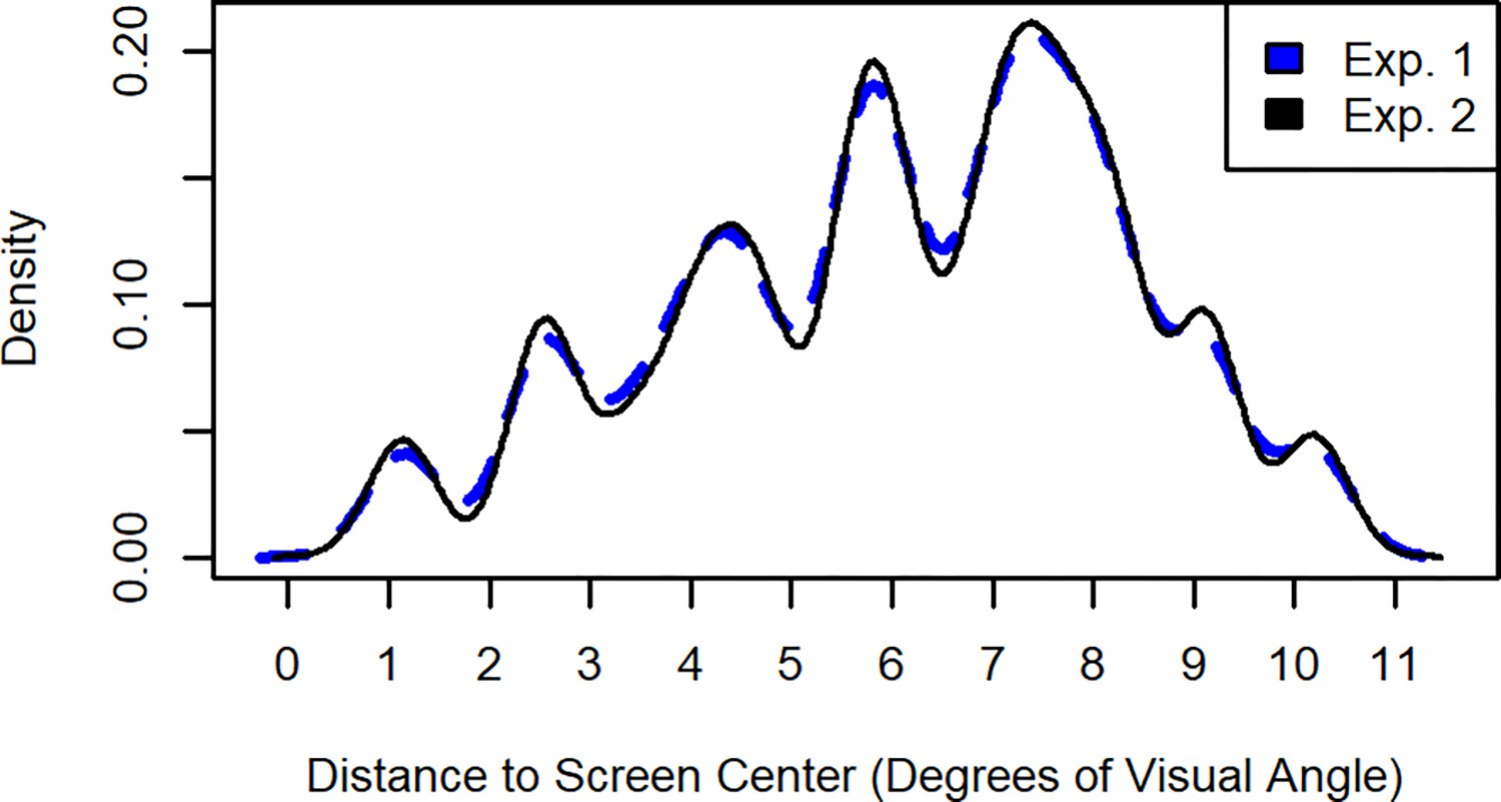
Density plot: Distance of probed object to the center of the screen (during encoding) in Experiments 1 and 2.

We fitted generalized linear mixed-effects models for each of the experimental conditions of our two experiments separately. In contrast to our main analyses that operated on an aggregated data set (sensitivity based on the mean hit rates and mean false alarm rates for each participant and each experimental cell), the generalized linear mixed-effect models were fit to the individual experimental trials, because this was required to include the distance of the probed object to the center of the screen as a fixed effect into the models. Thus, our dependent variable was the binary response (old location vs. new location) of participants to each trial and we fit those models using a binomial (logit) link function. As fixed effects, we included the main effects distance of the probed object to the center of the screen (defined as the distance in the encoding display in degree of visual angle; continuous predictor, see Fig 4 for a density plot showing its distribution in Experiments 1 and 2), configuration (global, single), location change (old location, location change), as well as all two-way interactions and the three-way interaction of those fixed effects. As random effect, we included the intercept for participants. We then evaluated the significance of the fixed effects (including their interactions) using Type II Wald chi-square tests.

**Fig 5:**
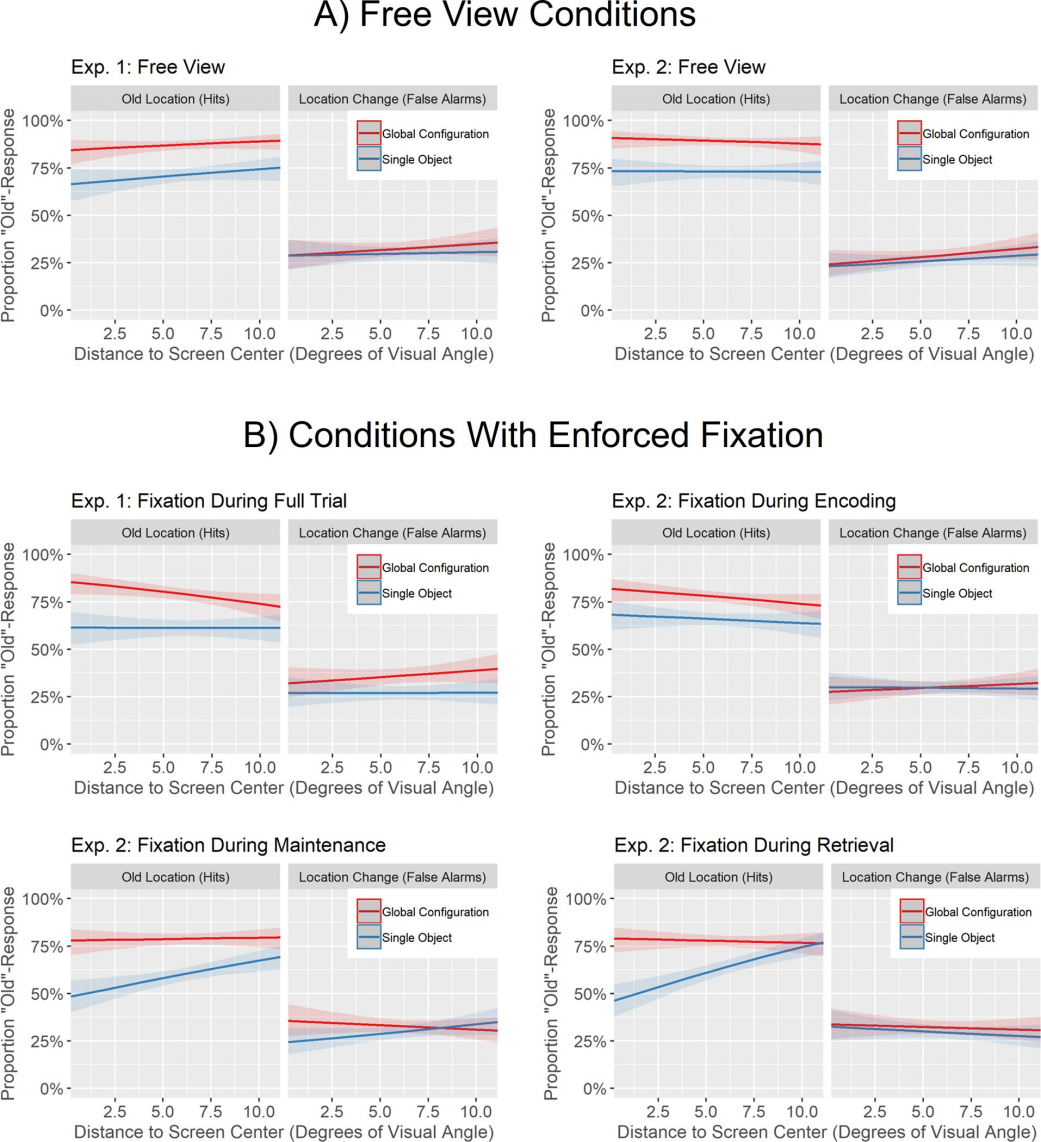
Predicted probabilities derived from the generalized linear mixed effect models to the experimental conditions of Experiments 1 and 2 to investigate the influence of the distance of the probed object to the center of the screen (during encoding) on hit rates and false alarm rates.

Consistent with the general benefit of spatial configurations on memory performance reported in our main analyses based on sensitivity, the interaction of configuration and location change was significant in all models (see Table 2 and Fig 5). That is, the presence of the global spatial configuration during retrieval resulted in a stronger increase of old response in trials with old location (hit rate) than in trials with location change (false alarm rate), reflecting participants increased ability in detecting location changes (hits minus false alarms) when the spatial configuration was present.

**Table 2:** Type II Wald chi-square tests used to evaluate the significance of the main effects and interactions of distance (D), configuration (C), and location change (L) in the generalized linear mixed effect models fitted to the experimental conditions of Experiments 1 and 2.

	D		C		L		D x C		D x L		C x L		D x C x L	
Condition	Χ^2^	*p*	Χ^2^	*p*	Χ^2^	*p*	Χ^2^	*p*	Χ^2^	*p*	Χ^2^	*p*	Χ^2^	*p*
Free View														
Experiment 1 (E1)	3.22	.073	**46.48**	**< .001**	**871.97**	**< .001**	0.15	.699	0.49	.484	**38.41**	**< .001**	0.08	.780
Experiment 2 (E2)	0.96	.328	**52.11**	**< .001**	**1108.30**	**< .001**	0.03	.863	2.23	.135	**44.64**	**< .001**	0.41	.524
Enforced Fixation														
Full Trial (E1)	0.16	.687	**90.14**	**< .001**	**603.67**	**< .001**	0.42	.518	2.69	.101	**10.89**	**.001**	2.90	.089
Encoding (E2)	0.63	.429	**20.15**	**< .001**	**731.07**	**< .001**	<0.01	.992	1.75	.186	**18.17**	**< .001**	0.76	.383
Maintenance (E2)	**4.91**	**.027**	**60.36**	**< .001**	**626.67**	**< .001**	**5.72**	**.017**	1.24	.265	**35.28**	**< .001**	<0.01	.958
Retrieval (E2)	2.30	.129	**35.41**	**< .001**	**670.54**	**< .001**	**4.34**	**.037**	**7.44**	**.006**	**17.56**	**< .001**	**6.67**	**.010**

Bold font indicates statistical significance (p < .05)

Regarding the influence of the distance of the probed object to the center of the screen on participants’ response pattern, this exploratory analysis revealed multiple insights (see Table 2 and Fig 5). First, when participants were free to move their eyes, there was no significant effect of distance on participants’ response pattern. Thus, distance had no baseline effect, such as objects in the center of the screen being remembered better, that we would need to consider when interpreting the results of the conditions with enforced fixation. Second, contrary to the assumption of covert visual attention supporting the encoding of object locations or the spatial configuration, there was also no significant effect nor significant interactions including distance for the condition with enforced fixation throughout the trial. This was the most informative experimental condition for this hypothesis, because overt attention was fixed to the center of the screen throughout the trial, causing the distance of the probed object to the center of the screen to correspond with the distance of the probed object to the fixation location. Third, there were differential effects of distance for the three enforced fixation condition of Experiment 2. For the condition with enforced fixation during encoding, distance did not significantly affect participants’ response pattern. For the condition with enforced fixation during maintenance, there was a significant main effect of distance as well as a significant interaction of distance and configuration. This interaction suggests that whereas distance had no influence on participants’ responses when the global spatial configuration was presented during retrieval, participants tendency in responding “new” increased the closer the probed object was to the center of the screen for the single object condition. For the condition with enforced fixation during retrieval, there was a significant main effect of distance, a significant two-way interaction of distance and configuration as well as a significant three-way interaction of distance, configuration and location change. Whereas participants’ responses where again unaffected by distance if the global spatial configuration was present during retrieval, participants were more likely to respond “new” to the probed object the closer the probed object was to the center of the screen during encoding for the single object condition, but only for old locations. Thus, participants location change detection performance (hits minus false alarms) decreased the closer the probed object was to the center of the screen for this single object condition. In this sense, lower distance might indeed have contributed to the configuration benefit if fixation was enforced during retrieval, but not by an increase in location change detection performance for the global configuration condition but by a decrease in location change detection performance for the single object condition. We further discuss the results of this exploratory analysis in the General Discussion.

## General discussion

We conducted two experiments investigating the influence of overt visual attention on the processing of spatial configurations in VSWM. As a proxy for the processing of spatial configurations, we used the well-documented configuration benefit, namely the higher performance in the detection of location changes of single objects under the presence of all encoded objects (i.e. spatial configuration) than the object probed alone [1–3]. We implemented three eye-movement conditions: free view, enforced fixation throughout the trial, and enforced fixation during specific task phases (i.e. encoding, maintenance, retrieval). In our experiments, we observed that the possibility of performing eye movements supported the configuration benefit. Interestingly, the processing of spatial configurations was reduced only in the condition that enforced fixation throughout the whole trial but not in conditions enforcing fixation during specific phases. That is, whereas configurational processing is supported by the performance of eye-movements, it does not rely on eye-movements occurring throughout a trial.

We identified two competing accounts regarding the processing of spatial configurations in VSWM in the literature, namely the snapshot account, which assumes a rather rigid, snapshot-like, representation of spatial configurations in VSWM [7, 17], and the flexible account, which focuses on hierarchical representations in VSWM with objects being organized in higher order hierarchical clusters [19–21]. Whereas shifting overt visual attention with eye-movements should impair the processing of spatial configurations based on the snapshot account [7, 24], the performance of eye-movements might support the representation of individual objects [8–10], and as a consequence also inter-object relations and spatial configurations based on the flexible account. Our results support the latter view, namely that eye-movements support rather than hinder the processing of spatial configurations. Thereby, our results support the flexible account, namely that spatial configurations are represented rather in the form of hierarchical representations based on the individual object representations than within a separate snapshot storage. The conclusion that spatial configurations are represented in a flexible form rather than as a rigid snapshot is also in line with recent evidence demonstrating that the representation of spatial configurations in VSWM can be updated based on retro cues and thus after encoding has already been completed [3–5].

For individual objects, previous research suggests a strong relationship between overt visual attention, covert visual attention, and VSWM [8–11, 33, 34]. More specific, the performance of eye movements towards objects is associated with respective shifts in covered visual attention [8, 9] as well as the allocation of VSWM resources [10]. Vice versa, VSWM is an important component of the eye movement system [12]. While most of the previous research investigated VSWM processes of single objects and their features, our results provide evidence for the role of eye-movements when processing inter-object relations in the form of spatial configurations in VSWM.

To our knowledge, this is the first study, which shows that limited non-availability of overt visual attention with regard to processing spatial configurations can be compensated via VSWM. That is, only enforcing fixation throughout a trial caused a significant reduction in the processing of spatial configurations. If the eyes were enforced to fixate during specific phases (i.e. encoding, maintenance, retrieval) only and free to move during the rest of a trial, no reduction in the configuration benefit was observed. Thus, eye movements do not support the processing of spatial configuration during either encoding, maintenance, or retrieval. Rather, it seems that single movements of the eyes might be sufficient to trigger the observed increase in configurational processing. While it is up to future research to identify the specific process underlying this increase in configurational processing, we speculate that processes related to trans-saccadic memory (TSM) and its interplay with VSWM might be involved. Based on TSM, observers establish correspondence between pre-saccadic and post-saccadic information, and TSM capacity is larger than or equal to VSWM capacity [35]. Therefore, it seems possible that TSM draws on inter-object relations such as spatial configurations, thereby also informing VSWM representations.

Our results are also consistent with previous research that demonstrated decreased change detection performance when participants were prevented from executing saccades to the object locations of interest due to enforced fixation [14, 15] or task-irrelevant eye-movements [13]. That is, we also observed that overall change detection performance decreased in all conditions that introduced enforced fixations, no matter whether fixation was enforced throughout a trial or at specific task phases. However, we think that it is important to distinguish between overall memory performance on the one hand and the configuration benefit defined as the difference in performance between global configuration and single object conditions on the other hand. Whereas overall task performance (especially in the single object condition) usually refers to participants’ general ability in memorizing independent object locations under specific conditions, the configuration benefit usually refers to the additional benefit provided by inter-object relations on memory performance. Regarding the configuration benefit, we found that only the complete enforcement but not partial enforcement of fixations caused a reduction in the processing of spatial configurations. Thus, being able to perform eye movements during at least some phases of a trial supports the processing of spatial configurations. It is important to note, however, that we observed a configuration benefit in all conditions. That is, even in conditions with enforced fixation throughout a trial, location change detection performance for single objects did benefit from the presence of the global spatial configuration of all encoded objects during retrieval. That is, despite this configuration benefit being lower with enforced fixations, it was still present. This suggests that observers also process spatial configurations without eye movements. This configurational processing is, thus, supported by eye movements but it does not depend on eye movements.

As an alternative interpretation of our results, one might argue that the requirement to conform to the fixation instruction in the enforced fixation conditions served as a dual task. Our results might, thus, be related to general dual-task interference rather than eye-movements. Although we did not include a control experiment with a dual task to rule out this explanation empirically, we do not believe that our central conclusions are affected by this alternative explanation for the following two reasons. First, our main conclusions are based on the influence of eye movements on the configuration benefit, namely the difference in performance between the global configuration and single object conditions. Whereas enforcing fixation throughout the trial resulted in a marked reduction of this configuration benefit in Experiment 1, enforcing fixation during a specific trial phase did not in Experiment 2. Because the enforced fixation conditions in Experiment 2 also required participants to conform with the eye-movement instructions throughout the trial (fixate in one phase but move eyes in other phases), the enforced fixation conditions of Experiments 1 and 2 should have caused comparable dual-task interference and thus the same influence on the configuration benefit in both experiments if our results were only due to dual-task interference, which was clearly not the case. Second, within the context of a related project investigating the representation of spatial configuration in VSWM [2], we performed a control experiment where participants performed the location change detection task either with or without articulatory suppression. Within this control experiment, we observed neither a main effect of articulatory suppression nor an interaction of the presence of articulatory suppression and the configuration benefit. This speaks against the idea that dual tasks generally interfere with the configuration benefit. Certainly, we cannot exclude the possibility that the overall drop in memory performance in the conditions with enforced fixation might be caused by the additional task demands introduced by the enforced fixation conditions, and thus this overall drop in memory performance with enforced fixation should be interpreted with care.

Within our present research, we were interested in investigating the role of shifts of overt visual attention on the processing of spatial configurations in VSWM. Therefore, we designed two experiments in which we compared free viewing conditions with conditions in which we enforced fixation throughout the trial (Experiment 1) or within specific trial phases (Experiment 2). We did, however, not control for shifts of covert visual attention. Thus, it remains possible that participants used shifts of covert visual attention to encode, maintain or retrieve information in our experiments. Previous research used dual tasks to direct participants’ covert visual attention to specific locations by requiring participants to constantly monitor those locations for changes [14, 15, 31]. It was found that enforcing covert visual attention to specific locations had similar effects as enforcing fixation [14, 15, 31], and thus it might sometimes be shifts of covert visual attention rather than eye-movements per se that underly the functional role of eye-movements [31]. Therefore, we consider it an encouraging route for future research to investigate the role of covert shifts of visual attention and to try to disentangle the contribution of eye-movements and covert shifts of visual attention on the processing of spatial configurations. This seems particularly interesting given prior research from our lab that showed that using retro cues to direct participants’ attention to parts of a spatial configuration during maintenance causes a reorganization of those configurations during maintenance [3–5], but that this retro-cue was no longer effective under conditions of enforced fixation [3].

With our exploratory analysis across Experiments 1 and 2, we tried to gain some exploratory insights on whether participants might have used shifts of covert visual attention to perform the location change detection task in our enforced fixation conditions. The condition with enforced fixation throughout the trial in Experiment 1 was the most informative condition for this analysis and it showed no influence of the distance of the probed object to the center of the screen during encoding on memory performance, which provides some tentative support for the idea that participants did not use shifts of their covert visual attention to perform the task during enforced fixation. The only conditions that showed some influences of the distance of the probed object to the center of the screen during encoding were the conditions with free view eye-movements during encoding and forced fixation during either maintenance or retrieval in Experiment 2, and this influence occurred only when the probed object was presented alone (without global configuration) during test. Participants might, thus, be very efficient in encoding the spatial configuration with free view eye-movements, but the performance of eye-movements might have caused them to spend more time on encoding the peripheral than central objects which in turn might have reduced location change detection performance for the central objects when presented alone. This might be particularly true because the objects were randomly positioned within a virtual square centered on the screen within our experiments, which caused more objects to be presented in the periphery than the center of the screen. Furthermore, our manipulation forced participants to look away from the center at least once during the free view encoding phase, and this may have further reinforced the tendency to preferentially encode peripheral objects. This interpretation is, however, very speculative and should be further investigated by future research.

In Experiment 2, there was a programming error that affected the free view encoding phases in the experimental blocks with enforced fixation during maintenance and enforced fixation during retrieval. Due to this error, the presentation time of the encoding phase was not prolonged by the presentation time of the blue cross that indicated participants to move their eyes away from fixation within this phase. Thus, participants viewed the encoding stimulus for a variable duration between 300 ms and 2000 ms instead of the fixed 2000 ms intended. In theory, this programming would have allowed participants to maintain fixation also during the encoding phase in those two experimental blocks. Given the data presented in S2 Appendix, we consider it unlikely that participants indeed used this fixation strategy for two reasons. First, because the blue cross indicating participants to move their eyes away from fixation was correctly implemented in all other conditions (and participants were instructed to move their eyes once it appears), participants likely learned that a trial with a blue cross does not continue without moving the eyes, and they then also implemented this strategy for the free view encoding phases (even though the experimental program did not enforce the eye movement by pausing the trial until an eye movement occurred). Second, if participants would have fixated instead of free viewed during those free view encoding phases, participants would likely have continued to fixate also in the following maintenance phase. However, the data in S2 Appendix shows that participants had a hard time to fixate in the maintenance phase after a free view encoding phase (67.7% of trials with a pause due to not adhering to the fixation instruction) and they had almost no issues to free view in the maintenance phase after a free view encoding phase (only 17.1% of trials with a pause due to not adhering to the free view instruction). This suggests that participants used a free view strategy already during the free view encoding phases in our Experiment 2 as intended.

## Conclusion

Participants utilized the ability of performing shifts of overt visual attention by eye movements to increase their memory performance. Experiment 1 demonstrated that eye movements support the configurational processing of object locations in memory. Whereas participants in Experiment 2 showed a lower overall performance in the conditions containing phases of enforced fixation, the size of the configuration benefit was similar across conditions. Thus, whereas the ability of performing shifts of overt visual attention by eye movements supports the processing of spatial configurations, this configurational processing does not rely on these shifts occurring throughout the task.

## Supporting information

S1 Appendix Fixation and saccade parameters for hit and false alarm trials in the free view conditions of Experiment 1 and 2. (PDF)

S2 Appendix Proportion of paused trials and pause durations for the three conditions with enforced fixation in Experiment 2. (PDF)
