# Supplementary material for: Processing spatial configurations in visuospatial working memory is influenced by shifts of overt visual attention
Source: PLoS One. 2023 Feb 9;18(2):e0281445. doi: 10.1371/journal.pone.0281445 (PMC9910631; doi:10.1371/journal.pone.0281445)
Supplement: S1 Appendix — (PDF) [file pone.0281445.s001.pdf]

S1 Appendix A

Within this appendix, we present the results of the preregistered analysis to compare fixation and saccade parameters between hit and false alarm trials in the free view conditions of Experiment 1 and 2 (see Table A.1). We used the high-speed event-detection algorithm provided by the IDF Event Detector (SMI, Version 3.0.20) with the following detection parameters: minimum duration of 22 ms, peak velocity threshold of 40°/s, and a minimum fixation duration of 50 ms. This analysis included all trials for which a maximum of 20% of the gaze samples were flagged as invalid by the eye-tracker and also all trials with a maximum response time of 10 s (93.39% of all trials included in this analysis for Experiment 1, and 92.92% of all trials included in this analysis for Experiment 2). For Experiment 1, there was no significant difference between hit and false alarm trials in the number of fixations, $t(55) = -0.63$, $p = .530$, the frequency of fixations (number of fixations divided by trial duration), $t(55) = 0.69$, $p = .490$, saccade amplitudes (saccade length in pixels), $t(54) = -0.52$, $p = .605$, nor the number of saccades, $t(55) = -0.16$, $p = .873$. For Experiment 2, there was also no significant difference between hit and false alarm trials in the number of fixations, $t(59) = -1.08$, $p = .283$, the frequency of fixations, $t(59) = 0.85$, $p = .398$, saccade amplitudes, $t(58) = -1.42$, $p = .161$, nor the number of saccades, $t(59) = -0.75$, $p = .455$.

Table A.1

Eye movement parameter means (SD) of the free view conditions for Experiments 1 and 2

Visual parameter	Hit trials	False alarm trials
Number of fixations (E1)	8.56 (4.21)	8.70 (4.17)
Frequency of fixations (E1)	1.32 (0.60)	1.30 (0.59)
Number of saccades (E1)	7.44 (4.52)	7.47 (4.33)
Saccade amplitude* (E1)	198.57 (48.82)	200.27 (50.30)

Number of fixations (E2)	9.16 (4.00)	9.40 (4.57)
Frequency of fixations (E2)	1.38 (0.58)	1.36 (0.62)
Number of saccades (E2)	8.00 (3.81)	8.15 (4.36)
Saccade amplitude* (E2)	199.93 (39.62)	204.58 (36.66)

* in pixel
