# Supplementary material for: Processing spatial configurations in visuospatial working memory is influenced by shifts of overt visual attention
Source: PLoS One. 2023 Feb 9;18(2):e0281445. doi: 10.1371/journal.pone.0281445 (PMC9910631; doi:10.1371/journal.pone.0281445)
Supplement: S2 Appendix — (PDF) [file pone.0281445.s002.pdf]

S2 Appendix B

Table B.1

Proportion of trials with at least one pause due to participants either not holding fixation or not moving the eyes away from fixation during the respective trial phase. Pause duration refers to the mean total duration of the pauses within a respective trial phase for each participant for those trials where at least one pause occurred.

Experimental Block (Fixation Phase)	Trial Phase	Proportion Trials With Pause (in %)		Mean Total Pause Duration (in ms)	
		M	*SD*	*M*	*SD*
Encoding	Encoding	33.7	18.2	506	214
	Maintenance	62.1	20.2	580	224
	Retrieval	11.8	13.8	476	225
Maintenance	Encoding	–[a]	–[a]	–[a]	–[a]
	Maintenance	67.7	24.9	523	306
	Retrieval	37.6	19.6	461	185
Retrieval	Encoding	–[a]	–[a]	–[a]	–[a]
	Maintenance	17.1	19.3	921	413
	Retrieval	49.5	25.3	522	261

[a] Due to a programming error, trial duration was not extended by the presentation time of the blue cross requesting participants to move their eyes away from fixation, and thus those pause values were also not logged and were not available for this exploratory analysis.
